# Deformation pattern and predictive value of right ventricular longitudinal strain in children with pulmonary arterial hypertension

**DOI:** 10.1186/s12947-016-0074-3

**Published:** 2016-07-29

**Authors:** Iolanda Muntean, Theodora Benedek, Mihaela Melinte, Carmen Suteu, Rodica Togãnel

**Affiliations:** 1 Clinic of Paediatric Cardiology, Institute of Cardiovascular Diseases and Transplantation, University of Medicine and Pharmacy, Tîrgu-Mures, Romania; 2 Clinic of Cardiology, University of Medicine and Pharmacy, Tîrgu-Mures, Romania; 3 Laboratory Department, Institute of Cardiovascular Diseases and Transplantation, Tîrgu-Mures, Romania; 4 Clinic of Paediatric Cardiology, Institute of Cardiovascular Diseases and Transplantation, University of Medicine and Pharmacy Tîrgu-Mures, 50 Gh Marinescu St, Tîrgu-Mures, 540136 Romania

**Keywords:** Right ventricle, Strain, Deformation pattern, Pulmonary arterial hypertension, Children

## Abstract

**Background:**

Right ventricular function has been identified as an important prognostic factor in children with pulmonary arterial hypertension. The aim of the study was to assess the deformation pattern and prognostic value of right ventricular longitudinal strain in children with pulmonary arterial hypertension.

**Methods:**

We prospectively evaluated 46 children (25 with pulmonary arterial hyperetension and 21 age and sex matched controls) using conventional and speckle-tracking echocardiography, brain natriuretic peptide levels and clinical status expressed by WHO functional class and 6-min walking test. According to the clinical status after 12 months of follow-up, the pulmonary arterial hypertension patients were divided in two groups: non-worsening (13) and worsening (12).

**Results:**

Right ventricular free wall longitudinal strain and strain rate were significantly lower in children with PAH compared with controls (−24.72 ± 3.48 vs −15.60 ± 3.40, *p* = 0.0001 and −1.44 ± 0.09 vs −1.09 ± 0.15, *p* = 0.0001, respectively). There was a more expressed decrease of basal than apical region of right ventricular free wall longitudinal strain/strain rate in pulmonary arterial hypertension patients compared with controls (strain: *p* = 0.0001 vs *p* = 0.07 and strain rate: *p* = 0.0001 vs *p* = 0.002). Comparing worsening and non-worsening pulmonary arterial hypertension patients there was a significant difference in Mid right ventricular free wall longitudinal strain (−14.00 ± 3.13 vs. −20.76 ± 4.62, *p* = 0.0001). In multivariable logistic regression analysis Mid right ventricular free wall longitudinal strain was an independent predictor of worsening in pulmonary arterial hypertension children (OR 0.45; 95 % CI: 0.21–0.96, *p* = 0.041). In ROC curve analysis a *cut-off* value of Mid right ventricular free wall longitudinal strain of −18.50 % predicted clinical worsening in pulmonary arterial hypertension children, with a sensitivity and specificity of 91.7 and 30.8 %, respectively (area under curve = 0.88 ± 0.06, 95 % CI: 0.75–1.00, *p* = 0.001).

**Conclusions:**

Two-dimensional speckle-tracking echocardiography is a complementary non-invasive tool for assessment of right ventricular function in children with severe pulmonary arterial hypertension, allowing also clinical prediction and segmental analysis of right ventricular myocardial performance in these patients.

## Background

Pulmonary arterial hypertension (PAH) is a rare disease in childhood that is related with significant morbidity and mortality. The most common types of PAH in pediatric population are PAH associated with congenital heart defects (chd-PAH) and idiopathic PAH (IPAH) [1–4], with a reported annual incidence rate of about 2.2 and 0.7 cases per million, respectively [5].

There are several clinical, laboratory and echocardiographic parameters that have been shown to be associated with higher risk of death in pediatric pulmonary hypertension, such as: Word Health Organization (WHO) functional class 3/4 [1, 4, 6], elevated levels of brain natriuretic peptide (BNP) [1, 7, 8] or evidence of right ventricular (RV) failure [1]. WHO functional class has been shown to correlate well with 6-min walk distance (6MWD) and hemodynamic parameters [1–3].

RV function has been identified as an important prognostic factor in patients with PAH regardless of the causal clinical entity [9, 10]. Therefore, accurate evaluation of RV function is crucial in the management of these patients. This can be challenging because of RV anatomy. In clinical practice, a qualitative subjective evaluation of the RV systolic function is performed routinely. The conventional echocardiographic parameters such as right ventricular fractional area changes (RV-FAC), tricuspid annular plane systolic excursion (TAPSE), right ventricular myocardial performance index (RV-MPI), tricuspid annular systolic velocity (S’) or RV systolic/diastolic (S/D) ratio have been validated until now in estimation of global RV performance in adults and in children as well [11–17]. The recently developed speckle-tracking based strain imaging has been reported in adult patients as a technique that allows a better quantitative assessment of regional myocardial motion and deformation in PAH patients [18, 19], however its usefulness in children has not been elucidated so far [14, 20, 21].

The aim of this study was to assess the deformation pattern and prognostic value of RV longitudinal strain in children with PAH.

## Methods

### Study population and grouping

This is a prospective clinical observational study. The study group consisted of forty six patients: twenty-five children with PAH and twenty-one age and sex matched healthy children as a control group (Table 1). All children underwent a complete physical examination, 6 min walking test, blood sampling for BNP serum level and complex echocardiographic examination.

**Table 1:** Clinical and laboratory (BNP level) characteristics of normal controls and PAH patients

Variable	Control (*n* = 21)	PAH patients (*n* = 25)	*P*
Age (y)	10.97 ± 3.48	11.07 ± 3.44	0.821
Male (%)	10 (47.61 %)	11 (44.00 %)	1.000
H (cm)	147.50 (133.25,156.75)	136 (119.00,147.00)	0.081
W (kg)	42.15 ± 18.15	29.95 ± 12.07	0.010
BSA (m2)	1.29 ± 0.36	1.05 ± 0.27	0.012
BMI (m/kg^2^)	18.31 ± 3.48	15.53 ± 2.97	0.016
6MWD (m)	480.35 ± 55.03	413.66 ± 75.31	0.001
SpO_2_ before 6MWT (%)	98.00 (95.20,98.90)	85.71 ± 9.52	0.0001
SpO_2_ after 6MWT (%)	98.00 (97.00,99.00)	75.80 ± 15.59	0.0001
HR before 6MWT (bpm)	86.15 ± 11.79	98.90 ± 14.38	0.190
HR after 6MWT (bpm)	109.20 ± 14.84	114.90 ± 16.09	0.246
WHO Functional class 1/2/3/4	NA	0/15/9/1 0/60 %/36 %/4 %	
BNP level (pg/ml)	9.00 (10.48,16.34)	25.85 (27.98,80.28)	0.005
PAH type			
Idiopathic	NA	5 (20 %)	NA
Ventricular septal defect	NA	12 (48 %)	NA
Atrioventricular septal defect	NA	5 (20 %)	NA
Truncus arteriosus communis	NA	3 (12 %)	NA
Medication no(%)			
Sildenafil	NA	5 (20 %)	NA
Bosentan	NA	13 (52 %)	NA
Combination (Sildenafil + Bosentan)	NA	7 (28 %)	NA

Data are presented as means ± SD or median (25th, 75th percentiles) or as numbers (percentages)

*BMI* body mass index, *BNP* brain natriuretic peptide, *BSA* body surface area, *H* height, *HR* heart rate, *PAH* pulmonary arterial hypertension, *SpO*_*2*_ oxygen saturation, *W* weight, *6MWD* 6-min walk distance

PAH was defined as mean pulmonary arterial pressure ≥ 25 mmHg at rest [1]. The PAH group consisted of children with idiopathic PAH (*n* = 5) or PAH associated with congenital heart defects, with tripartite right ventricle. The associated congenital heart defects in the study population were: ventricular septal defect (*n* = 12), complete atrioventricular septal defect (*n* = 5) and truncus arteriosus communis (*n* = 3), all with pulmonary hypertensive vascular disease (with right-to-left or bidirectional intracardiac shunting)*.* All PAH patients in the present study were on pulmonary vasodilator medication in monotherapy or combined therapy (Table 1). Further we split the PAH patient group in nonworsening and worsening group depending on the clinical evolution and WHO functional class after a follow-up period of 12 months.

The control group consisted of children with no history of cardiovascular disease, normal electrocardiography and echocardiography recordings, who were investigated for chest pain or fatigability and in whom the presence of any heart disease was rulled out.

#### Exclusion criteria

Patients older than 18 years old, those with PAH secondary to left heart disease or single heart (Nice, 2013) [1] and patients with rhythm disturbances were excluded from the study.

### Echocardiographic image acquisition

The children were scanned with iE33 (Philips Medical Systems, Best, Netherlands) ultrasound system using an S5-1 transducer, by a single sonographer. At least three consecutive beats were stored in cine-loop format. Frame rate (60 to 100 frames per second), depth and sector width were adjusted in order to obtain accurate speckle-tracking analysis of the RV myocardial deformation.

Conventional echocardiographic parameters such as RV-FAC, TAPSE, RV-MPI, S’, RV S/D ratio, pulmonary ejection time (Tej), LV eccentricity index (LV-EI) and mean pulmonary arterial pressure (PAPm) were measured as recommended by current guidelines [12, 13, 22–24]^.^

### Two-dimensional speckle-tracking analysis

**Fig. 1:**
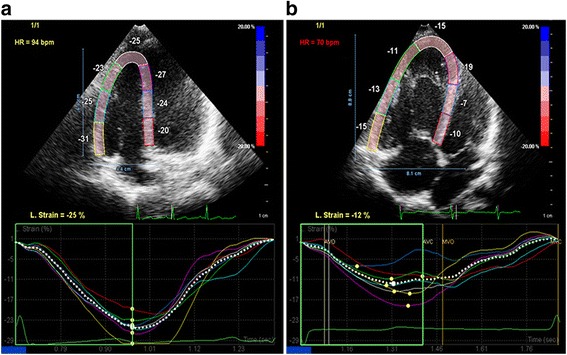
2D speckle-tracking strain image of RV. LpsS in different segments. **a** control group; **b** PAH group. LpsS, longitudinal peak systolic strain; PAH, pulmonary arterial hypertension; RV, right ventricle

Speckle-tracking analysis of the RV was performed offline, from the RV-focused 4-chamber view images, using commercially available software (QLAB 10, Philips Medical Systems, Best, Netherlands). The RV endocardial border has been automatically generated by the software and than manually adjusted if necesarry. A seven-segment model was created according to the software: three at the RV free wall, three at the interventricular septum (IVS) and one at the apex of the RV. Also, speckle-tracking RV longitudinal strain and strain rate curves were automatically generated for every segment (Fig. 1) [13, 17–20].

Longitudinal peak systolic strain (LpsS) and longitudinal peak systolic strain rate (LpsSR) were measured in the basal, midventricular and apical segments of the RV free wall, IVS and RV apex, respectivelly. RV free wall LpsS and LpsSR were calculated as the average of the three segments, and conventionally are presented as a negative value [13, 17–20].

### Six-minutes walking test (6MWT)

6MWT was done corresponding to the American Thoracic Society for adults [25]. Children were instructed to walk as fast as possible on a flat ground for 6 min. Before and after the test, heart rate and transcutaneaus oxygen saturation (SpO_2_) were measured by pulse oximeter. 19 controls and 23 PAH patients were able to perform 6MWT.

### Functional class

The functional class was established according to WHO class system [26].

### Measurement of BNP

All blood samples were taken on the day of echocardiographic examination. The samples were centrifugated, and plasma was stored at −70 °C until analysis was performed. BNP was assayed on an automatic immunoassay analyzer (Architect i1000SR, Abbott Diagnostics).

### Follow-up

All PAH children were evaluated after 12 months for the occurence of the primary end point, clinical worsening. Clinical worsening was defined as increase in WHO functional class.

### Statistical analysis

Statistical analysis was performed using SPSS version 20 (IBM SPSS STATISTICS 20). Data were labelled as nominal or quantitative variables. Nominal variables were expressed as numbers or percentages. Quantitative variables were tested for normality of distribution using Kolmogorov-Smirnov test and were expressed by mean ± standard deviation or median and percentiles (25;75 %), whenever appropriate. Differences between the mean or median between two groups were analysed using the t-test, Mann-Whitney test or chi-test when appropriate. Differences between three groups with normally distributed variables were analysed using ANOVA test with Bonferroni posthoc processing. Multivariate analysis was carried out using logistic regressions. We use as dependent variable the groups of PAH children (worsening and non-worsening). The covariates were Mid RV free wall LpsS, Mid RV free wall LpsSR, Apical RV free wall LpsS, BNP and LV EI. Receiver-operating characteristic curves were constructed, and areas under curve were calculated. Sensitivities and specificities were determined for the ability to identify worsening PAH children. Intraobserver reliability was assessed after reexamination by the same examiner 2 weeks after the initial examination, by intraclass correlation coefficient. A *p*-value of < 0.05 was considered statistically significant.

## Results

### Clinical and laboratory characteristics

Twenty-five consecutive children with PAH and twenty-one age and sex matched normal children were studied. The main clinical characteristics of the study groups are presented in Table 1. Children with PAH had lower body weight, body surface area and body mass index, probably explained by the impact of disease on nutritional status of PAH patients. The 6MWD, mean SpO2 before and after 6MWT were significantly lower in patients with PAH compared with controls. More than half of the PAH children were in WHO functional class 2. Plasma BNP level was significantly higher in patients with PAH compared with controls.

### Conventional echocardiographic data

The conventional echocardiographic parameters of the two groups are compared in Table 2. RV-MPI, RV S/D ratio, indexed RA area, LV EI were significantly higher and RV-FAC, TAPSE, Tej and S’ were significantly lower in PAH group than in control group, indicating RV function impairment in patients with PAH. There was no difference between the groups regarding left ventricular ejection fraction (LV-EF).

**Table 2:** Comparison of conventional echocardiographic parameters between normal controls and PAH patients

Variable	Control (*n* = 21)	PAH (*n* = 25)	*P*
PAPm (mmHg)	NA	62.83 ± 17.42	NA
TR (mmHg)	NA	93.76 ± 24.52	NA
sRV pressure (mmHg)	NA	104.80 ± 30.82	NA
Pericardial effusion	NA	7/25 (28 %)	NA
RV-FAC (%)	45.36 ± 5.60	37.24 ± 13.12	0.008
Indexed RV end-diastolic area (cm^2^)	9.82 (9.23,12.05)	15.88 ± 5.07	0.0001
Indexed RV end-systolic area (cm^2^)	5.79 ± 1.18	10.22 ± 4.69	0.0001
RV MPI	0.12 ± 0.07	0.32 (0.24,0.51)	0.0001
TAPSE (cm)	2.19 ± 0.47	1.79 ± 0.60	0.019
RV S/D ratio	0.82 (0.81,1.03)	1.30 ± 0.33	0.0001
Indexed RA area (cm^2^)	8.41 ± 1.32	11.87 (9.53,14,57)	0.0001
Tej (ms)	319.01 ± 33.45	273.05 ± 31.04	0.0001
LV-EI	1.02 (0.97,1.04)	1.33 (1.21,1.56)	0.0001
S’ (cm/s)	13.62 ± 1.78	11.75 ± 3.53	0.026
LV-EF	69.00 ± 4.71	71.20 ± 9.88	0.330

Data are presented as means ± SD or median (25th, 75th percentiles)

*FAC* fractional area change, *LV-EF* left ventricular ejection fraction, *LV-EI* left ventricular eccentricity index, *MPI* myocardial performance index, *PAH* pulmonary arterial hypertension, *PAPm* mean pulmonary arterial pressure, *RA* right atrium, *RV* right ventricle, *S’* tricuspid annular systolic velocity, *S/D ratio* systolic/diastolic ratio, *sRV pressure* systolic right ventricular pressure, *TAPSE* tricuspid annular plane systolic excursion, *Tej* pulmonary ejection time, *TR* tricuspid regurgitation

### Two-dimensional speckle-tracking echocardiography

**Fig. 2:**
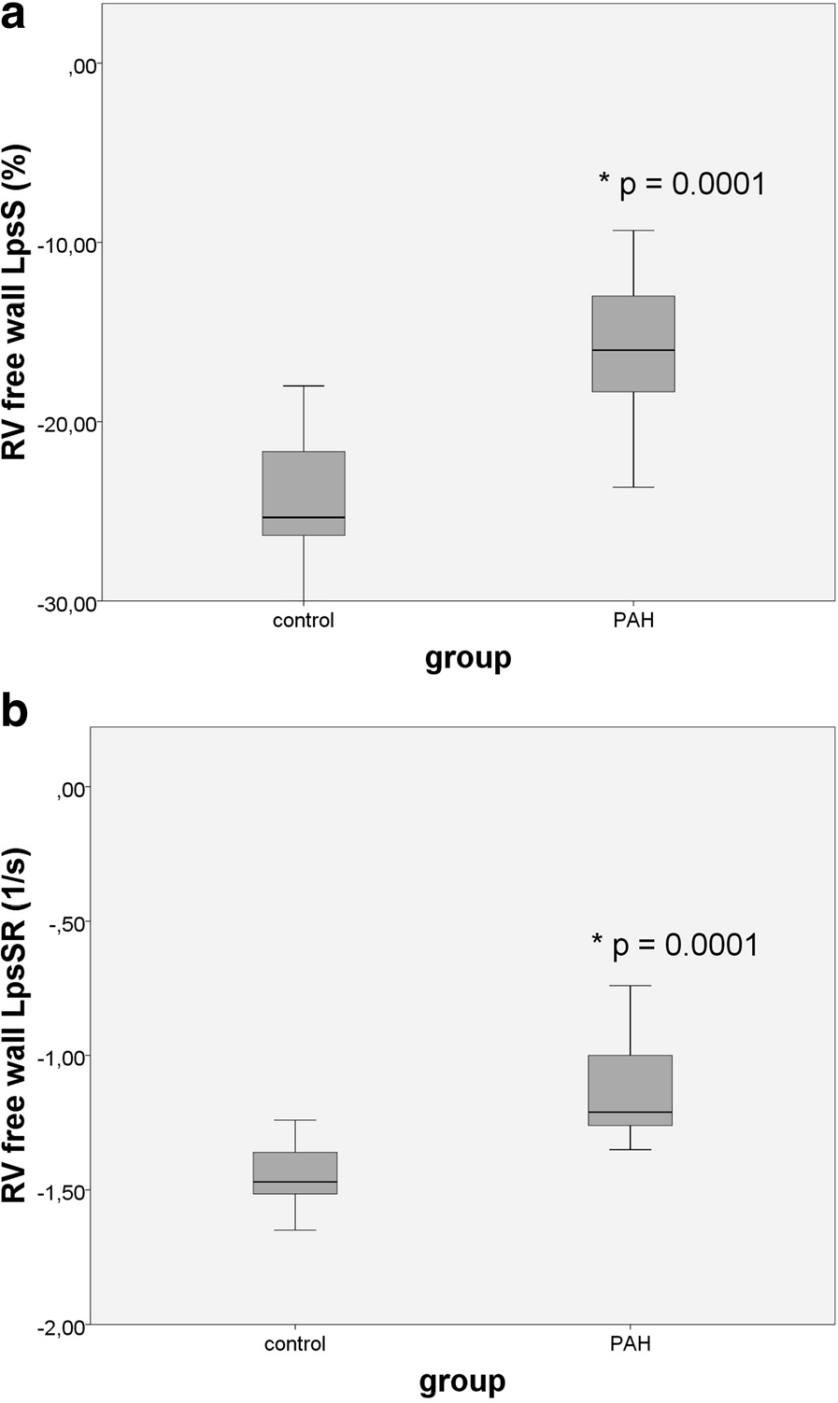
Comparison between controls and PAH patients regarding 2D strain indices of RV free wall; **a** LpsS **b** LpsSR. c, control; LpsS, longitudinal peak systolic strain; LpsSR, longitudinal peak systolic strain rate; PAH, pulmonary arterial hypertension; RV, right ventricle; *p < 0.05

**Fig. 3:**
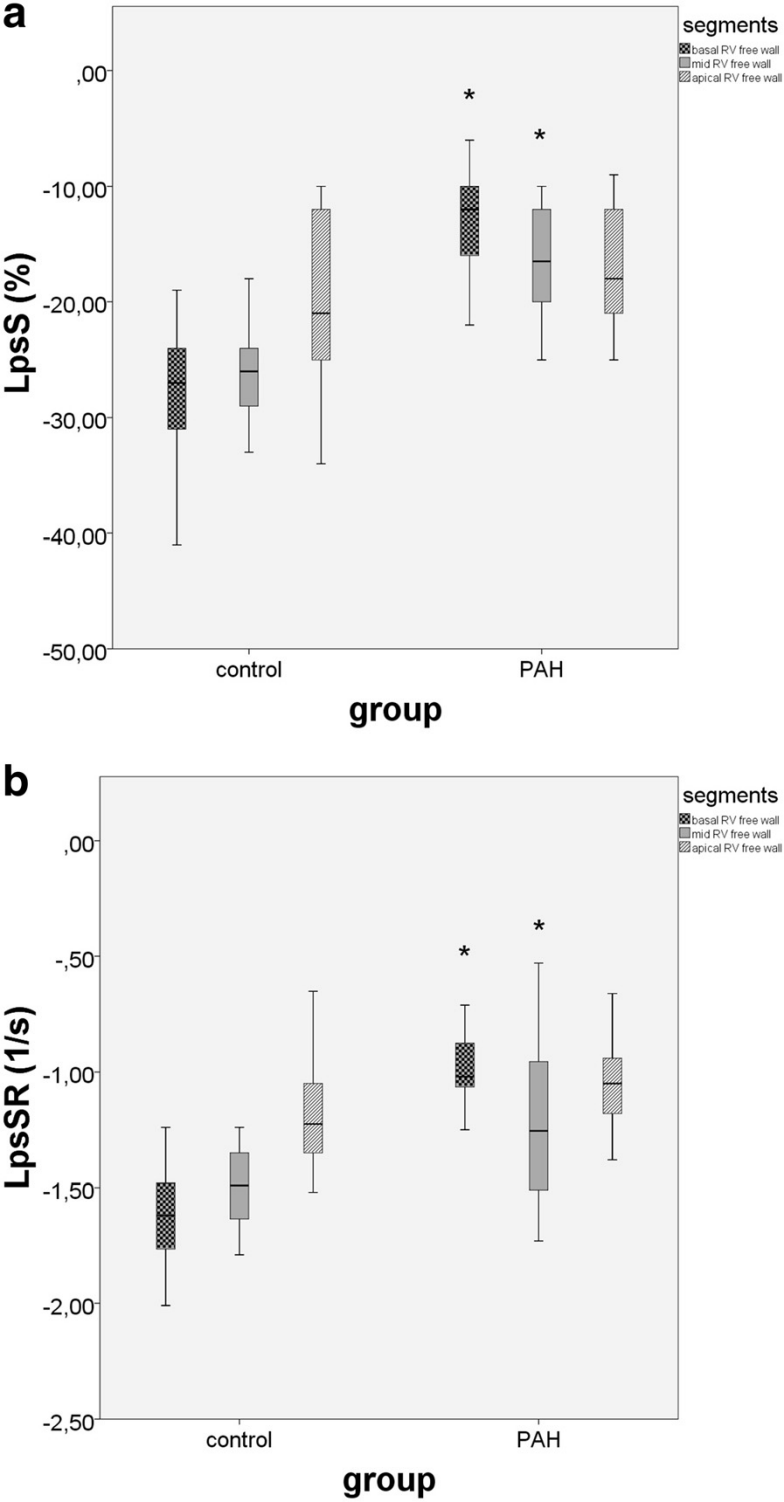
Comparison between control group and PAH patients regarding 2D strain measurements of RV free wall segments: basal, mid and apical. **a** LpsS; **b** LpsSR. LpsS, longitudinal peak systolic strain; LpsSR, longitudinal peak systolic strain rate; PAH, pulmonary arterial hypertension; RV, right ventricle; * PAH vs control group, *p* < 0.05

Table 3 shows the speckle-tracking parameters of control group and PAH patients. *RV free wall* LpsS and LpsSR were significantly lower in PAH patients than in controls (Fig. 2a and b). RV free wall LpsS and LpsSR were higher in basal segment (−28.19 ± 5.47 and −1.61 ± 0.19) in comparison with the apical segment (−20.00 ± 7.58 and −1.21 ± 0.18) in healthy children, sugesting a *base-to-apex gradient*. This base-to-apex gradient was not observed in PAH children (Fig. 3a and b). However, in PAH patients there was a more expressed decrease of LpsS and LpsSR in the basal region of the RV free wall compared with the apical one (Fig. 3a and b). Because of lack of the basal septal region in most of chd-PAH patients, LpsS and LpsSR could not be accurately measured in this segment.

**Table 3:** Comparison of longitudinal strain indices in different RV segments between control group and PAH children

Variable	Control group (*n* = 21)	PAH children (*n* = 25)	*p*
*RV-LpsS (%)*			
RV free wall	−24.72 ± 3.48	−15.60 ± 3.40	0.0001
Basal RV free wall	−28.19 ± 5.47	−12.80 ± 4.26	0.0001
Mid RV free wall	−26.00 ± 4.45	−17.52 ± 5.20	0.0001
Apical RV free wall	−20.00 ± 7.58	−16.48 ± 4.96	0.077
Apex	−23.66 ± 5.95	−17.76 ± 4.47	0.0001
IVS			
Apical IVS	−27.33 ± 5.59	−19.00 (−20.50,−16.00)	0.0001
Mid IVS	−24.38 ± 5.34	−17.36 ± 5.70	0.0001
Basal IVS	−17.00 ± 4.18	NA	NA
*RV-LpsSR(s* ^*−1*^ *)*			
RV free wall	−1.44 ± 0.09	−1.09 ± 0.15	0.0001
Basal RV free wall	−1.61 ± 0.19	−1.00 ± 0.15	0.0001
Mid RV free wall	−1.50 ± 0.17	−1.23 ± 0.31	0.001
Apical RV free wall	−1.21 ± 0.18	−1.04 ± 0.16	0.002
Apex	−1.28 ± 0.26	−1.09 ± 0.19	0.009
IVS			
Apical IVS	−1.30 (−1.60,−1.22)	−1.21 ± 0.26	0.016
Mid IVS	−1.45 ± 0.32	−1.11 ± 0.30	0.001
Basal IVS	−1.14 ± 0.24	NA	NA

Data are presented as means ± SD or median (25th, 75th percentiles)

*c* control, *IVS* interventricular septum, *LpsS* longitudinal peak systolic strain, *LpsSR* longitudinal peak systolic strain rate, *NA* no available, *PAH* pulmonary arterial hypertension, *RV* right ventricle

### Predictive value of strain imaging

After 12 months of follow-up, twelve children with PAH (48 %) presented clinical worsening with augmentation of the WHO functional class.

Table 4 show comparison of clinical, BNP level, conventional echocardiographic parameters, and longitudinal strain values (LpsS and LpsSR) in different RV segments in two PAH groups: non-worsening and worsening. Although, all parameters are impaired in worsening group compared with non-worsening group, there is significant difference only in case of BNP level, LV-EI, Mid RV free wall LpsS, Apical RV free wall LpsS, Mid RV free wall LpsSR.

**Table 4:** Comparison of clinical, BNP level, conventional echocardiographic parameters, and longitudinal strain values (LpsS and LpsSR) in different RV segments in two PAH groups: non-worsening and worsening

Variable	Non-worsening PAH children (*n* = 13)	Worsening PAH children (*n* = 12)	*p*
6MWD (m)	416.41 ± 68.72	380.90 ± 104.00	0.341
SpO2 before 6MWT (%)	86.00 ± 11.11	84.30 ± 7.83	0.689
SpO2 after 6MWT (%)	77.41 ± 18.22	74.50 ± 11.53	0.667
WHO Functional class	2.38 ± 0.50	2.50 ± 0.67	0.631
BNP_0_ level (pg/ml)	30.53 ± 37.03	82.01 ± 74.80	0.039
PAPm (mmHg)	61.53 ± 18.25	64.36 ± 17.13	0.701
TR (mmHg)	91.23 ± 31.74	96.50 ± 14.05	0.602
sRV pressure (mmHg)	98.61 ± 40.41	111.50 ± 14.05	0.297
Pericardial effusion	2/13 (8 %)	5/12 (20 %)	0.200
RV-FAC (%)	41.07 ± 15.10	33.08 ± 9.51	0.131
RV MPI	0.26 (0.24,0.39)	0.35 (0.29,0.59)	0.129
TAPSE (cm)	1.93 ± 0.59	1.64 ± 0.61	0.247
RV S/D ratio	1.24 ± 0.28	1.36 ± 0.39	0.367
Indexed RA area (cm^2^)	12.63 ± 3.33	15.35 ± 15.70	0.547
Tej (ms)	280.50 ± 31.95	264.98 ± 29.18	0.219
LV-EI	1.31 ± 0.17	1.56 ± 0.26	0.010
S’ (cm/s)	12.83 ± 3.34	10.58 ± 3.50	0.114
LV-EF	71.76 ± 11.07	70.58 ± 8.86	0.771
*RV-LpsS (%)*			
RV free wall	−17.38 ± 2.51	−13.66 ± 3.24	0.004
Basal RV free wall	−12.69 ± 4.64	−12.91 ± 4.01	0.899
Mid RV free wall	−20.76 ± 4.62	−14.00 ± 3.13	0.0001
Apical RV free wall	−18.69 ± 4.00	−14.08 ± 4.92	0.017
Apex	−19.15 ± 4.25	−16.25 ± 4.37	0.106
IVS			
Apical IVS	−19.69 ± 5.49	−19.00 (−22.00,−13.00)	0.337
Mid IVS	−18.30 ± 4.76	−16.33 ± 6.63	0.399
Basal IVS	NA	NA	NA
*RV-LpsSR(s* ^*−1*^ *)*			
RV free wall	−1.16 ± 0.12	−1.01 ± 0.13	0.008
Basal RV free wall	−1.00 ± 0.19	−1.00 ± 0.09	0.956
Mid RV free wall	−1.37 ± 0.27	−1.07 ± 0.29	0.013
Apical RV free wall	−1.11 ± 0.12	−0.97 ± 0.16	0.090
Apex	−1.15 ± 0.19	−1.04 ± 0.18	0.166
IVS			
Apical IVS	−1.21 ± 0.32	−1.20 ± 0.20	0.896
Mid IVS	−1.17 ± 0.27	−1.05 ± 0.34	0.372
Basal IVS	NA	NA	NA

Data are presented as means ± SD or median (25th, 75th percentiles) or as numbers (percentages)

*BNP* brain natriuretic peptide, *FAC* fractional area change, *IVS* interventricular septum, *LpsS* longitudinal peak systolic strain, *LpsSR* longitudinal peak systolic strain rate, *LV-EF* left ventricular ejection fraction, *LV-EI* left ventricular eccentricity index, *MPI* myocardial performance index, *NA* no available, *PAH* pulmonary arterial hypertension, *PAPm* mean pulmonary arterial pressure, *RA* right atrium, *RV* right ventricle, *S’* tricuspid annular systolic velocity, *S/D ratio* systolic/diastolic ratio, *SpO*_*2*_ oxygen saturation, *sRV pressure* systolic right ventricular pressure, *TAPSE* tricuspid annular plane systolic excursion, *Tej* pulmonary ejection time, *TR* tricuspid regurgitation, *6MWD* 6-min walk distance

**Fig. 4:**
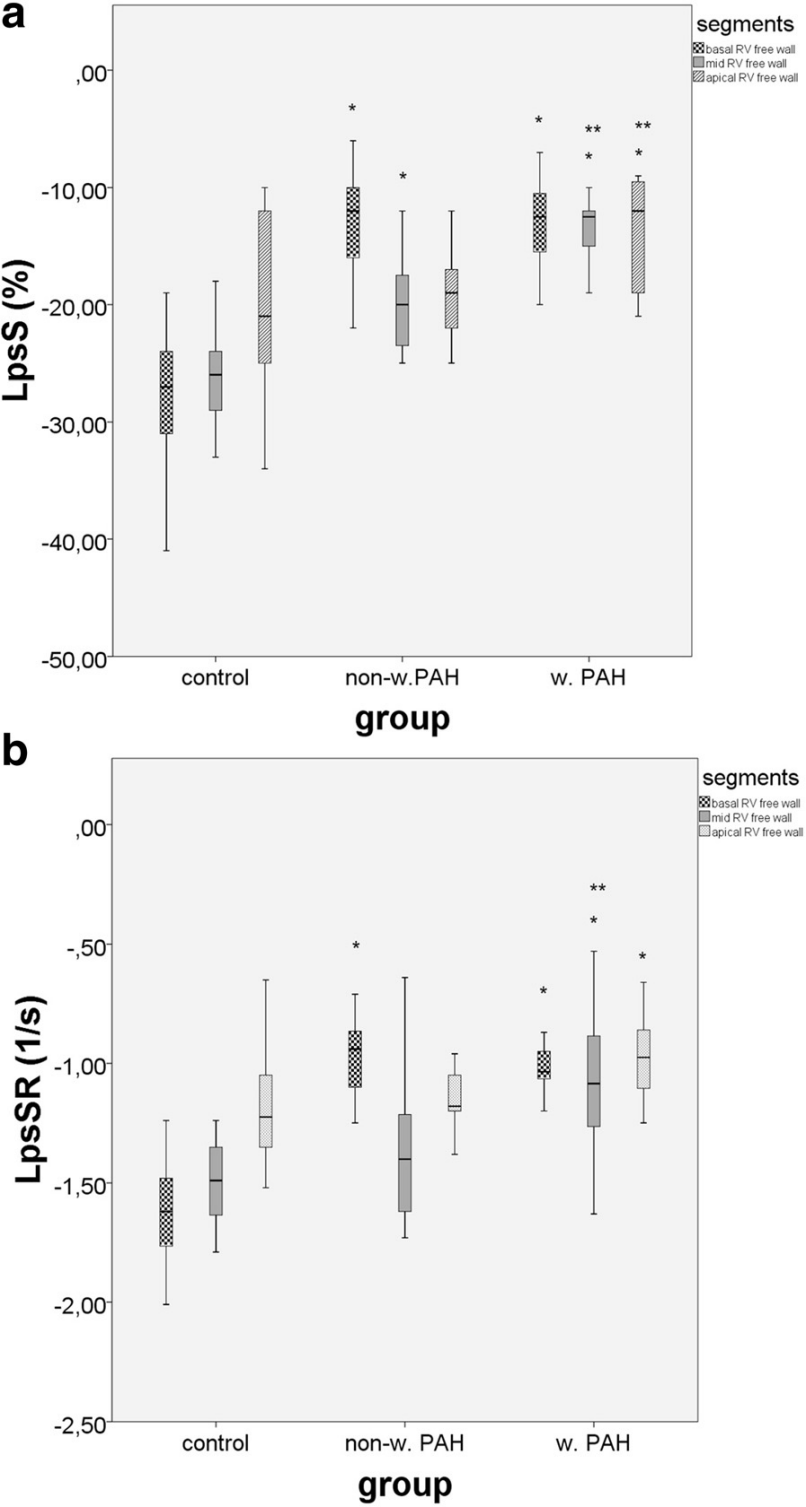
Comparison between control group, non-worsening PAH patients and worsening PAH patients regarding 2D strain measurements of RV free wall segments: basal, mid and apical. **a** LpsS; **b** LpsSR. LpsS, longitudinal peak systolic strain;LpsSR, longitudinal peak systolic strain rate non-w, non-worsening; PAH pulmonary arterial hypertension; RV, right ventricle; w, worsening; *non-w. or w. PAH vs control; ** w. vs non-w. PAH patients; *p* < 0.05

Figure 4 show comparison of longitudinal strain values (LpsS and LpsSR) in different RV segments in three study groups: non-worsening PAH children, worsening PAH children and controls. All, but the apical RV free wall LpsS were significantly lower in non-worsening PAH children compared with controls (Fig. 4). On the other hand, all RV free wall segments LpsS decreased significantly in worsening PAH children compared with controls. Comparing worsening and non-worsening PAH groups, only Mid and Apical RV free wall LpsS decreased significantly. Longitudinal strain rate was significantly lower in Basal RV free wall segment among non–worsening PAH children compared with normal controls. Only Mid RV free wall LpsSR decreased significantly among worsening compared with non-worsening PAH children.

We performed a multivariable logistic regression analysis, considering the worsening versus non-worsening PAH children as dependent variables and the BNP, LV-EI, Mid RV free wall LpsS, Apical RV free wall LpsS and Mid RV free wall LpsSR, as covariates. After controlling for these covariates, multivariate analysis showed that Mid RV free wall LpsS was an independent predictor of worsening in PAH children (Table 5).

**Table 5:** Multivariable logistic regression analysis for prediction of worsening in PAH patients

Variable	Multivariate		
	Odds ratio	95 % CI	*p*
BNP	1.027	0.995–1.059	0.097
LV EI	0.082	0.000–49.076	0.444
Mid RV free wall LpsS	0.458	0.217–0.967	0.041
Mid RV free wall LpsSR	0.084	0.000–62.398	0.462
Apical RV free wall LpsS	1.003	0.694–1.448	0.989

*BNP* brain natriuretic peptide, *LpsS* longitudinal peak systolic strain, *LpsSR* longitudinal peak systolic strain rate, *LV-EI* left ventricular eccentricity index, *PAH* pulmonary arterial hypertension, *RV* right ventricle

**Fig. 5:**
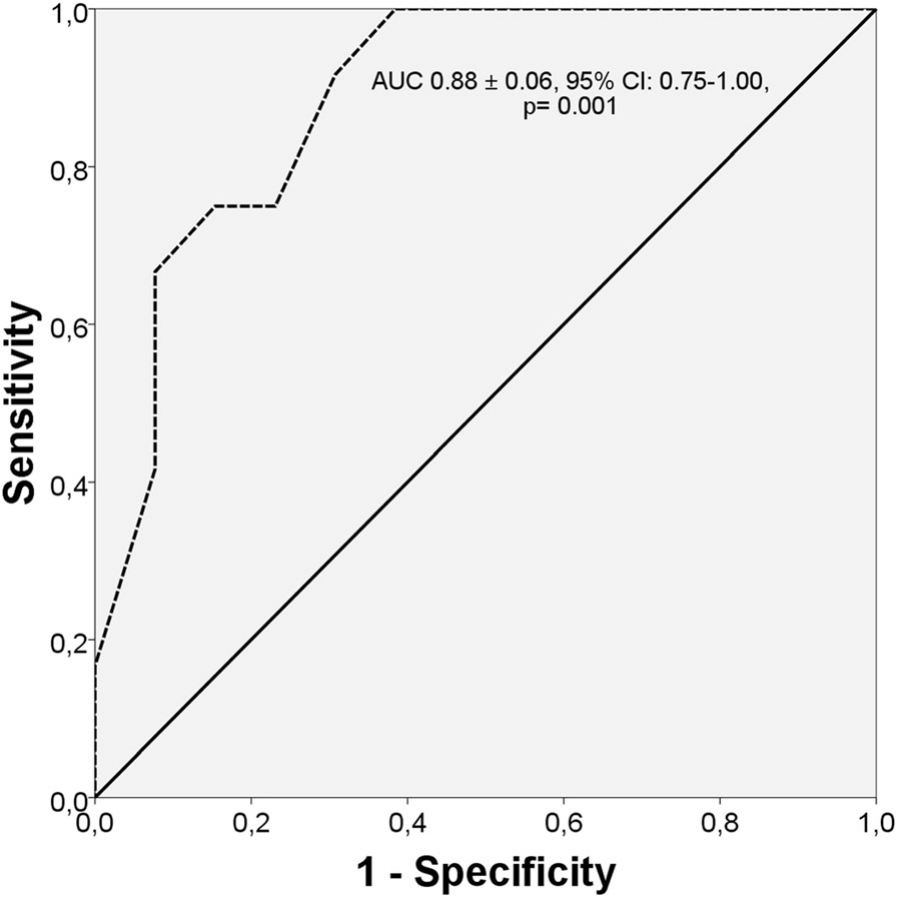
Receiver-operator characteristic analysis. The optimum cut-off value for Mid RV free wall LpsS to predict clinical worsening was −18.50 %; the sensitivity and specificity were 91.7 and 30.8 %. The area under curve was 0.88 ± 0.06 (95 % CI: 0.75–1.00, *p* = 0.001)

In ROC curve analysis, a cut-off value of Mid RV free wall LpsS of −18.50 % predicted clinical worsening in PAH children, with a sensitivity and specificity of 91.7 and 30.8 %, respectively. (area under curve = 0.88 ± 0.06, 95 % CI: 0.75–1.00, *p* = 0.001, Fig. 5.)

### Intraobserver reliability

Intraobserver reliability assessed by intraclass correlation coefficient was 0.90; 95 %CI (0.76-9.96), *p* = 0.0001.

## Discussion

RV function is an important prognostic factor in PAH [1], however its echocardiographic assessment is challenging in adults and in children as well, due to the particular anatomy of the RV [13, 14, 17, 19, 20, 22]. Although, *conventional echocardiographic parameters* are largely used in clinical practice even in children, they have some intrinsic limitations [13–17]. These indices miss information regarding regional variation in function.

The present study has some important findings: 1) children with PAH seem to have a *deterioration pattern* of regional longitudinal deformation of RV: there is an initial decrease of strain/strain rate in the basal region followed by the mid/apical region 2) Mid RV free wall LpsS has predictive value in PAH children.

### RV strain in children

Two-dimensional (2D) *speckle-tracking* echocardiography has been recently introduced for assessment of myocardial performance. Several previous reports have shown its accuracy and feasibility to assess RV function in adults [18, 19, 27]. Only a few study have used this technique to measure regional RV deformation in PAH children [14, 17, 20, 21]. In this study, we evaluated RV longitudinal deformation pattern in PAH children compared with normal controls.

We found an average value of −24.72 ± 3.48 for RV free wall LpsS and −1.51 ± 0.23 for LpsSR in *normal children*, comparable with those reported in the literature [28, 29]. The slightly different values between studies could be explained by the use of different equipment and software [30]. Also, we found a base-to-apex gradient of RV segmental longitudinal strain indices in normal controls. Levy et al. reported this pattern in healthy adults and children as well [29].

Furthermore, our results show that RV free wall longitudinal strain indices (LpsS and LpsSR) are significantly lower in children with severe PAH compared with normal controls. However, it is important to underline that myocardial deformation is influenced by loading condition. Simon et al. revealed that, RV longitudinal strain can be decreased in both PAH patients with RV dysfunction and in PAH patients with preserved RV function. However, in the first situation RV strain was found to be much lower than in the second one [31, 32]. On the other hand, strain rate, representing the shortening velocity per fibre length, can describe more accurately the RV dysfunction. Ferferieva et al. demonstrated in an experimental study on mice, that strain rate correlates with the intrinsic contractile status of the myocardium, and is less influenced by changes in cardiac load [33]. Edvardson et al. revealed the clinical feasibility of strain rate imaging in quantifying regional systolic function [34].

### RV regional deformation pattern in children with PAH

The RV has a complex anatomy, and can be divided in three distinct parts: smooth inlet portion, trabeculated apical portion and outlet portion. The RV free wall is composed by longitudinal fibers in the subendocardial layer, more developed in healthy subjects, and circumferential fibers in the superficial layer [35]. This explains why longitudinal shortening is the main component of RV pump function in healthy people [36].

We found a non–homogeneous decrease of the longitudinal strain indices in different segments of RV free wall in PAH patients, with loss of the base-to-apex gradient compared with normal controls. Consistent with the prior studies on pulmonary hypertension [29], our study showed impaired basal longitudinal strains in all PAH children. It has been demonstrated that the inlet portion is a greater contributor to right ventricular systolic function than the apical or outlet portion [37, 38]. We suppose that this could explain the greater decrease of strain and strain rate in PAH patients in the basal region of the RV free wall as compared with the mid or apical one.

Furthermore, we found a significant decrease of Mid and Apical RV free wall strain indices in worsening group as compared to non-worsening group. Mauritz et al. reported that in RV the longitudinal shortening reduction starts at the basal level and continue to decrease in other regions until a lower limit is reached. Also, they suggested that a further reduction of RV function is due to loss of transverse shortening with leftward septal displacement [39].

In conclusion, our findings suggest a deterioration *pattern of regional longitudinal deformation* of RV free wall myocardium in PAH children. There is an initial decrease of strain in the basal region; a further decrease in mid and apical region precedes clinical worsening in PAH children. However, these findings have to be validated in larger studies.

### Predictive value of RV regional deformation in children with PAH

The predictive value of RV LpsS has been evaluated in adult patients with PAH. Haeck et al. demonstrated that RV global longitudinal strain ≥ −19 % is associated with a 3-fold risk of all-cause mortality [40]. In a study conducted on 575 adult patients with PAH, Fine et al. showed that RV free wall systolic strain predicted clinical outcome independent of other conventional echocardiographic parameters in adults [41]. Okumura et al. showed that RV global longitudinal > −14 %, could predict transplantation-free survival in children with idiopathic pulmonary arterial hypertension [21].

However, the predictive value of RV regional deformation in PAH children was not studied so far. We found that Mid RV free wall strain indices are significantly lower in worsening group as compared to non-worsening group. Furthermore, we showed that Mid RV free wall LpsS is an independent predictor of worsening in PAH children and a *cut-off* value of −18.50 % is associated with clinical worsening in these patients. According to our study, Shehata et al. demonstrated by MRI, in a study conducted on PAH patients, that regional (basal and mid) longitudinal free wall deformation can already be affected at the time that RV function is still normal, implying that regional deformation could detect early RV dysfunction in PAH [42]. According to our knowledge this is the first study that describes the predictive value of RV regional deformation in children with PAH.

### Limits of the study

The limitation of the study is that we did not compare our results with those obtained by magnetic resonance imaging (MRI), which is considered the “gold standard” method in RV function assessment. However, previous studies have validated the use of speckle-tracking derived strain against sonomicrometry [43] and magnetic resonance imaging [44]. An other limitation is the small number of the patients included in the present study. Further research with a larger number of children are needed to confirm these findings.

## Conclusion

Two-dimensional speckle-tracking echocardiography is a complementary non-invasive tool for assessment of right ventricular function in children with severe pulmonary arterial hypertension, allowing clinical prediction and segmental analysis of right ventricular myocardial deformation in these patients.

## Abbreviations

6MWD, 6-min walk distance; BNP, brain natriuretic peptide; chd, congenital heart defects; chd-PAH, pulmonary arterial hypertension associated with congenital heart defect; FAC, fractional area change; IVS, interventricular septum; LpsS, longitudinal peak systolic strain; LpsSR, longitudinal peak systolic strain rate; LV-EF, left ventricular ejection fraction; LV-EI, left ventricular eccentricity index; MPI, myocardial performance index; PAH, pulmonary arterial hypertension; PAPm, mean pulmonary arteial pressure; RA, right atrium; RV, right ventricle; S/D ratio, systolic/diastolic ratio; S’, tricuspid annular systolic velocity; SpO_2_, oxygen saturation; sRV pressure, systolic right ventricular pressure; TAPSE, tricuspid annular plane systolic excursion; Tej, pulmonary ejection time; TR, tricuspid regurgitation; WHO, World Health Organization
